# Design of a multi-site multi-state clinical trial of home monitoring of chronic disease in the community in Australia

**DOI:** 10.1186/1471-2458-14-1270

**Published:** 2014-12-15

**Authors:** Branko G Celler, Ross Sparks, Surya Nepal, Leila Alem, Marlien Varnfield, Jane Li, Julian Jang-Jaccard, Simon J McBride, Rajiv Jayasena

**Affiliations:** DPAS FlagshipeHealth Research Program, Cnr Vimiera and Pembroke Roads, PO Box 76, Epping NSW 1710, 2122 Marsfield, NSW Australia; CSIRO eHealth Research Program, Marsfield, NSW Australia

**Keywords:** Telehealth, Home telehealth, Home telemonitoring, Chronic disease management, Experimental protocol, BACI design, Case matched control design

## Abstract

**Background:**

Telehealth services based on at-home monitoring of vital signs and the administration of clinical questionnaires are being increasingly used to manage chronic disease in the community, but few statistically robust studies are available in Australia to evaluate a wide range of health and socio-economic outcomes. The objectives of this study are to use robust statistical methods to research the impact of at home telemonitoring on health care outcomes, acceptability of telemonitoring to patients, carers and clinicians and to identify workplace cultural factors and capacity for organisational change management that will impact on large scale national deployment of telehealth services. Additionally, to develop advanced modelling and data analytics tools to risk stratify patients on a daily basis to automatically identify exacerbations of their chronic conditions.

**Methods/Design:**

A clinical trial is proposed at five locations in five states and territories along the Eastern Seaboard of Australia. Each site will have 25 Test patients and 50 case matched control patients. All participants will be selected based on clinical criteria of at least two hospitalisations in the previous year or four or more admissions over the last five years for a range of one or more chronic conditions. Control patients are matched according to age, sex, major diagnosis and their Socio-Economic Indexes for Areas (SEIFA). The Trial Design is an Intervention control study based on the Before-After-Control-Impact (BACI) design.

**Discussion:**

Our preliminary data indicates that most outcome variables before and after the intervention are not stationary, and accordingly we model this behaviour using linear mixed-effects (lme) models which can flexibly model within-group correlation often present in longitudinal data with repeated measures. We expect reduced incidence of unscheduled hospitalisation as well as improvement in the management of chronically ill patients, leading to better and more cost effective care. Advanced data analytics together with clinical decision support will allow telehealth to be deployed in very large numbers nationally without placing an excessive workload on the monitoring facility or the patient's own clinicians.

**Trial registration:**

Registered with Australian New Zealand Clinical Trial Registry on 1^st^ April 2013. Trial ID: ACTRN12613000635763

## Background

In industrialized nations approximately 70-78% of healthcare budgets are spent on the management of chronic disease or its exacerbation [1–3] and as the population ages the burden of chronic disease increases and places healthcare budgets under increasing strain.

A strong primary health care system has been acknowledged as critical to the sustainability of health care systems both in developing and industrialised nations and it has emerged as a recurrent theme in recent years [4–6]. The management of chronic disease, much of which could occur in home and community settings, often unnecessarily burdens hospital-centric public health system. As a consequence policy makers and health service managers seek innovations that deliver the same or improved health services using proportionately fewer resources.

Telehealth services have been demonstrated internationally to be one such innovation [7, 8], but there are low levels of evidence from Australian studies [9]. This study will evaluate whether the introduction of in-home telemonitoring services to the management of chronic disease in the community reduces patient use of the health system and improves healthcare outcomes and their quality of life.

We will also explore the extent to which real-time risk stratification of these patients is of value to health professionals and the issues and challenges in deploying telemonitoring services in the community.

Telehealth and telecare technologies and services for the management of chronic disease at home and in the community have been of intense interest in developed western economies because of unprecedented growth rates of the aged population and increasing morbidity as population ages. These factors place unsustainable stress on established health care services, and will result in increasing deficits in clinical human resources, expanding disease management programs and patient demand for greater self-management.

Telehealth services, delivered through home tele-monitoring, have been demonstrated to deliver cost effective, timely and improved access to quality care [7, 9–12]. They also reduce social dislocation and enhance the quality of life and the sustainability of these communities by allowing chronically ill and aged members to stay in their homes and communities longer.

However experience in Australia with the deployment of Telehealth services is extremely limited [13], with most deployments of small scale and lacking detailed analysis of key success factors such as:

-  Health care outcomes
-  Health economic benefits
-  Impact on clinical work force availability and deployment
-  Human factors (acceptability, usability by patients, carers, nurses, GPs and administrators)
-  Workplace culture
-  Capacity for organisational change management and business processes

The development of a robust business case and business models for large scale commercial deployment of Telehealth services, based on reliable socio-economic evidence, is therefore essential if these services are to be deployed nationally to mitigate the escalating costs of health service delivery and the increasing deficit in clinical work force.

This trial will seeks to create a robust evidence base for these key success factors and demonstrate an effective and scalable model for an internet-enabled Telehealth services in Australia. Armed with the insights provided by this evidence base, policy makers may have much of the data they require to implement funding models and create a sustainable Telehealth services sector in Australia.

Despite large national investments in health IT, very little policy work has been undertaken in Australia in deploying Telehealth in the home as a solution to the increasing demands and costs of managing chronic disease. In contrast in the UK, the first report from the Department of Health (DH) on this subject was published in 2000 [14] and many others have followed since [15–17]. The DH’s Preventative Technology Grant (PTG) from 2006–08 provided £80 M to local authorities and their partners for investment in assistive technology [18] and most recently £31 m of funding for a Whole System Demonstrator (WSD) program which had Telehealth as an integral part for the management of long-term conditions [11, 19].

The Litan report [7] provides a comprehensive review of the international evidence that Telehealth services for the management of chronic disease can reduce admission to Accident and Emergency (A&E) from between 20-60%. In one report quoted, Strategic Healthcare Programs, LLC (2004), Physiological monitoring of Heart and lung disease and diabetes reduced A&E visits by 49% for Congestive Heart Failure (CHF) patients, 66% for Chronic Obstructive Pulmonary Disease (COPD) patients, and 83% for diabetes patients.

Most recently the Whole System Demonstrator [11] Headline Findings released by the UK Department of Health in December 2011, demonstrated;

-  15% reduction in A&E Visits
-  20% reduction in emergency admissions
-  14% reduction in elective admissions
-  14% reduction in bed days
-  8% reduction in tariff costs and
-  45% reduction in mortality rates

This was the largest randomised control trial of telehealth and telecare in the world, involving 6191 patients and 238 GP practices across three sites, Newham, Kent and Cornwall. Three thousand and thirty people with one of three conditions (diabetes, heart failure and COPD) were included in the telehealth trial [19].

The most large scale example of Telehealth use is in the US by the Veterans Health Administration (VHA). VHA mainstreams clinical care within its Coordinated Care and Home Telehealth (CCHT) project [12]. Routine analysis of data obtained for quality and performance purposes from a cohort of 17,025 CCHT patients shows the benefits of a 25% reduction in numbers of bed days of care, 19% reduction in numbers of hospital admissions, and mean satisfaction score rating of 86% after enrolment into the program. VHA’s experience is that an enterprise-wide home telehealth implementation is an appropriate and cost-effective way of managing chronic care patients in both urban and rural settings.

Most recently [20] the US Department of Veterans Affairs announced that 690,000 US veterans received care in the 2014 fiscal year via telehealth, with 2 million telehealth visits scheduled. That means that 12 percent of all veterans enrolled in VA programs received telehealth care of some kind in 2014.

### Aims and objectives

This study was designed with the aim of demonstrating how telehealth services for chronic disease management in the community can be deployed nationally in Australia in a range of hospital and community settings and to develop advanced modelling and data analytics tools to risk stratify patients on a daily basis to automatically identify exacerbations of their chronic conditions.

The following research questions will be addressed;

-  Effect of telemonitoring on health service utilisation
  - Unscheduled visits to hospital, visits to GPs and Nurse visits
  - Cost and frequency of consultations, laboratory tests and other clinical procedures
-  Effect of telemonitoring on patients outcomes
  - Quality of life, progression of chronic condition, wellbeing, medication adherence
-  Service implementation and deployment
  - Existing model of care, service design, adoption and appropriation
-  User experience and service implementation
  - Satisfaction, useability, acceptance, workload, anxiety and strain among study participants including health professionals, administrators, patients and carers

-  Service implementation issues
  - How the new home monitoring service is implemented at each site. What impact has this had on the process and outcomes of normal care delivery?
  - How are existing service practices evolving as a result of the new service?
  - What can be learnt from different implementation approaches?
-  Cost effectiveness analysis
  - Analysis of reductions/increases in costs borne by patients as a result of telehealth
  - Analysis of reductions/increases in costs borne by the commonwealth and on-the-ground service providers for patients as a result of the deployment of telehealth services

The Project Objectives are to:

-  Demonstrate and document how telehealth services can be successfully deployed across Australia, by piloting services in five different settings across five states with a range of health service provider’s, including Local Health Districts, Medicare Locals and not for profit community organisations. This will be demonstrated by deploying and demonstrating the operation of Telehealth monitoring in a multi-site multi-state case matched control trial (Before-After-Control-Impact (BACI) design) of chronically ill patients living in their own homes in the community. This has never previously been attempted in Australia.
-  Provide the clinical and health economic evidence on how Telehealth services can be scaled up nationally to provide an alternative cost effective health service for the management of chronic disease in the community.
-  Provide evidence that at home telemonitoring has the potential to reduce unscheduled admissions to Accident and Emergency (A&E) compared to the control group.
-  Provide evidence for an impact on hospital admissions, mortality, clinical events and symptoms and improvements in functional measures and patients' and carers’ experiences with care.
-  Evaluate health economic benefits
-  Evaluate impact on clinical work force availability and deployment
-  Evaluate impact of human factors (acceptability, usability by patients, carers, nurses, GPs and administrators, impact on workplace culture)
-  Evaluate impact of workplace culture
-  Evaluate impact of organisational change management and business processes
-  Develop a new evidence based data analytical technique for the risk stratification of patients’ health status daily and demonstrate that this facilitates the management of large numbers of patients by orchestrating an optimal and timely allocation of resources to avoid unnecessary hospitalisation

For each of the above objectives, operation of the trial at five different sites representing a range of different models for the management of chronic disease in the community will allow the identification and analysis of site specific differences in workplace culture, organisational change management and staff and management capabilities that contribute to differences in measured health, social and economic outcomes.

## Methods/Design

Immediately following granting of funds for the project in December 2013, Ethics Approval was sought and granted by the CSIRO CAFHS Human Research Ethics Committee, on the 25^th^ March 2013 (approval # 13/04). Subsequently, ethics approval was also obtained from health authorities at each of the test sites as well as from the Commonwealth Department of Health and Ageing and the Department of Human Services. The latter two were necessary in order to be granted access to national patient data stored in the Medicare MBS and PBS archives.

**Figure 1:**
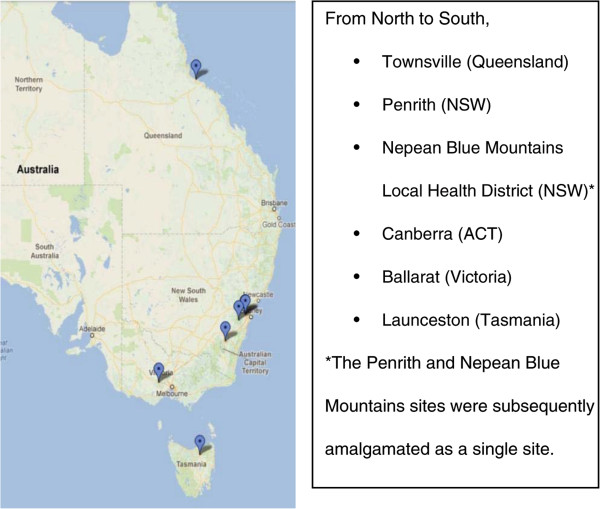
**Trial sites along Eastern seaboard of Australia and in Tasmania.**

The trial sites are located over widely dispersed regions of the Eastern Seaboard of Australia as shown in Figure 1. They were selected on the basis of three criteria, (i) early participation in the rollout of the National Broadband Network, (ii) geographical location and demographics and (iii) variations in models of care used to manage Chronic disease to be generally representative of the variety of models of care for the management of chronic disease existing in Australia.

### Organisation and management

Establishing an appropriate Governance model for managing such a complex project is critical in order to comply with the requirements of the National Statement on Ethical Conduct in Human Research (2007) [21], the specific requirements of multiple Human Research Ethics Committees and the statutory requirements of the Therapeutic Goods Administration regarding the use of medical devices for monitoring health status.

**Figure 2:**
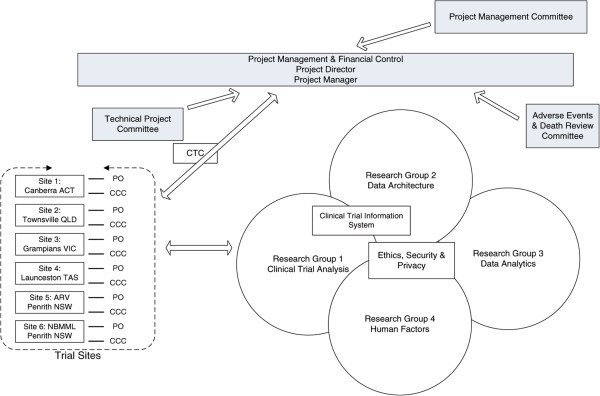
**Project organisation and management structure.** CTC – Clinical Trial Coordinator. PO –Project Officer and CCC – Clinical Care Coordinator at each trial site.

The Organisational structure shown below in Figure 2 was established in April 2013. Clinical groups meet on a weekly basis and are chaired by the Project Manager or the Clinical Trial Coordinator. The four research teams also meet weekly to monitor progress against project milestones. The Project Management Committee meets monthly to monitor and review progress of the project against its stated aims and objectives. The Management Committee is Chaired by the Project Director and includes representatives from each site as well as two clinicians, one representing the interests of General Practice and the other, Chairing the Adverse Events and Death Review committee which meets whenever necessary.

### Selection of trial participants

Since random selection of patients was not possible because of small sample sizes and the initial requirement that Test patients be selected from areas connected to the NBN, a **B**efore **A**fter Control **I**ntervention (BACI) design [22] was adopted that foregoes assumptions of normality. The BACI paired samples design provides greater control over confounding variables, increases the power of the study and improves the chances of finding a significant result with a smaller number of samples if the impact is relatively small.

The research protocol requires the recruitment of 75 participants at each of the five sites to achieve a total sample size of 375. Of these 125 are Test patients and 250 are Control patients. At each site 25 participants are allocated to the intervention, with 50 remaining control participants receiving normal care as per their site’s existing model of care.

Eligible candidates are identified primarily by searching the local hospital patient administration system (PAS) for patients who satisfy the eligibility criteria described in Table 1. Some candidates were also identified by site clinical staff familiar with their medical history. Typically between 250 and 300 eligible patients are identified by searching the hospital records of the major community hospital in the region. Candidates are eligible to participate in the study if they meet all inclusion and none of the exclusion criteria listed in Table 1 and become participants on the signing of informed consent in the presence of an independent witness.

**Table 1:** **Clinical criteria for eligibility**

Criteria	Type	Description
Age	Inclusion	50 years old and over at consent.
Cognitive capacity	Inclusion	Abbreviated Mental Test (AMT) [23] score >7.
Unplanned acute admissions	Inclusion	A rate of unplanned acute admission with the required principal diagnosis code(s) indicated below:
		a) ≥2 in the last 12 months, or
		b) ≥4 in the previous 5 years.
ICD-10-AM principal diagnosis code(s) for each unplanned acute admission	Inclusion	Code(s) for each unplanned acute admission indicate a diagnosis for one or more of the following chronic conditions:
		a) Chronic Obstructive Pulmonary Disease (J41 – J44, J47 and J20, with secondary diagnosis of J41-J44, J47),
		b) Coronary Artery Disease (I20 – I25),
		c) Hypertensive Diseases (I10 – I15, I11.9. Note: Hypertensive Heart Failure (I11.0) is included in Congestive Heart Failure),
		d) Congestive Heart Failure (I11.0, I50, J81),
		e) Diabetes (E10 - E14),
		f) Asthma (J45).
Unsuitable conditions	Exclusion	The study team considers the presence of the following conditions to be unsuitable for participation in the study:
		a) Any form of cancer,
		b) Any neuromuscular disease
		c) Any psychiatric conditions.
Care team	Inclusion	The eligible patients must be under the care of any of the following:
		a) General Practitioner
		b) Community Nurse
Care programs	Inclusion	Participation in one of the following government care programs:
		a) Commonwealth Chronic Disease Management
		b) Commonwealth Coordinated Veterans’ Care Program
		c) NSW Connected Care Program
Unsuitable care programs	Exclusion	Participation in one of the following government care programs:
		a) Commonwealth Extended Aged Care in the Home

For the purposes of our study unplanned admissions are all admissions other than:

1. Admissions from the waiting list (including both the surgical list and the medical waiting list);
2. Admissions listed as "regular same day planned admissions" which are admissions that are intended regular and planned same‒day admissions for an on‒going phase of treatment, such as renal dialysis or chemotherapy.

Following the signing of a consent form and completion of the Entry Questionnaire, Test patients are connected to the internet and supplied with the TMC Telemonitoring system and trained on its use by the Project Officer (PO) at each site. Their vital signs and questionnaire responses are subsequently monitored on a daily basis by the Clinical Care Coordinator (CCC). On site visits and technical support as well as the obtaining of Consent and the administration of Exit questionnaires are the responsibility of the PO.

Control patients also complete the Entry questionnaire but otherwise continue to receive normal care. For each intervention participant, as many as six control candidates are automatically case matched on gender, age, chronic condition and Socio-Economic Indexes for Areas (SEIFA) [24]. On their consent the two closest matching control candidates commence as participants in the study. The remaining four candidates are held in reserve. Table 2 below demonstrates the case matching process.

**Table 2:** **Example of case matching of Control patients with Test patients**

Test/control	Age	Gender	Major diagnosis	Seifa ^1^ index for postcode	Strength of match (perfect match = 0)
Test	54	M	COPD	1023	
Control	56	M	COPD	1025	1.68^2^
Control	54	F	HD	1022	2.16^3^
Weights	0.2	1	1	0.16	

^1^SEIFA 2011 Socio-Economic Indexes for Areas [24]. SEIFA provides measures of socio-economic conditions by geographic area.

^2^|54-56| × 0.2 + 1 × 0 + 1 × 0 + |1023-1015| × 0.16 = 1.68.

^3^|54-54| × 0.2 + 1 × 1 + 1 × 1 + |1023-1022| × 0.16 = 2.16.

Generally, the closer the match the greater the likelihood of finding a significant result with a smaller number of samples if the impact is relatively small.

Ideally, as many as four matches are sought for each Test patient, and the closest match is then selected as the case matched control for that Test patient. In many cases only one acceptable match may be available.

Following a change of Government in Australia, the requirement to connect patients to the high speed National Broadband Network was removed and as consequence, approximately half of our trial patients will be connected to a variety of NBN, ADSL, ADSL2 and VDSL network services with a minimum level of service of 2Mbps download and 1Mbps upload. Those on the NBN service were guaranteed a download speed of 25Mbit/sec and an upload speed of 5Mbit/sec.

### Subject enrollment and consent

Eligible patients are initially contacted by the Trial PO and given a detailed information sheet outlining the project. Confirmation of willingness to participate is via a consent document witnessed by a person independent of the project. Upon consenting the patient is administered a comprehensive Point of Entry Questionnaire as described in Table 3 using OpenClinica [25], the world's first commercial open source clinical trial software for Electronic Data Capture (EDC) and Clinical Data Management (CDM), as the data depository and data management system. Processes are then put in place to connect the telemonitoring system and to train the patient on its use. A Quality Control process operates for one week to ensure that patient training is adequate and the quality of the data recorded is acceptable.

**Table 3:** **Key elements of the Entry and Exit Questionnaires**

Section	Source/questionnaire
1-3	CSIRO Standard Screening Medical Questionnaire [29] + additional trial specific questions
	Selected questions from Living with Diabetes Study [30]
	Selected questions from Fat and Fibre Barometer [31]
4	Active Australia [32]
5	Kessler 10 [33]
6	Dimensions from HeiQ (Living with and managing medical conditions) [34]
7	EuroQol EQ-5D [35]
8	Dimensions from HeiQ (Social Isolation) [34]
9	Morisky Medication Adherence [36]

Once each Test patient is consented, an automated case matching process is carried out as already described.

### Selection of Tele-monitoring service provider

CSIRO undertook a comprehensive and independent Technology Assessment process to select the telehealth service provider for this project. The selection committee was chaired by the Leader of the Health Services Theme, and comprised six members including partner representatives.

From an initial review of companies operating in the Australia market, six were selected for further consideration based on length of presence in Australia and experience with supporting telehealth services in a trial environment. These were requested to provide detailed information on their at home tele-monitoring equipment and ultimately three were interviewed and asked to provide a physical demonstration of their equipment. Criteria for selection included the following;

Range of vital signs measurements available, patient Video conferencing and messaging capability, Clinical Questionnaires specific to patient condition, ability to view vital signs data in raw signal form, quality/ease of use of patient user interfaces, quality/ease of use of clinician user interfaces, expert system for daily patient risk profiling, high quality video for delivery of Educational/training material, multi language capability, quality standard and data accessibility, regulatory compliance (TGA, CE Mark, FDA, ISO13485), capability to export data from the system, opportunity to partner with the provider to trial new tools, algorithms and analytic results within their system, evidence of capability to deploy and service equipment in the field, including patient training, remote maintenance etc., availability of local R&D support, quality of support staff and management, evidence of experience in deploying telehealth services, cost of Equipment/Services and data storage location.

Based on a comparative analysis of the criteria identified above, Telemedcare Systems Pty Ltd [26] was selected as the telehealth service. Critical attributes identified were (i) meeting all TGA, FDA and CE Marking regulatory requirements, (ii) commercial presence in Australia since 2006 and proven track record in supporting telehealth projects in Australia and the UK, (iii) Local customer support and software and hardware R&D capability, (iv) advanced clinical capability including spirometry, ecg and auscultatory NIBP and strong local R&D support and (v) ability to view all recorded traces for purposes of improved diagnosis and quality control particularly in patients who may have cardiac arrhythmia.

**Figure 3:**
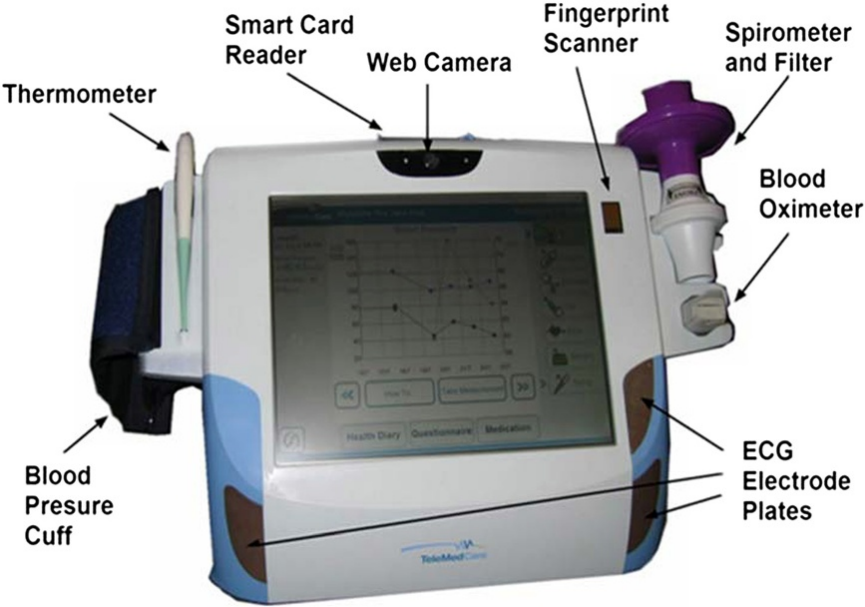
**Telemedcare Clinical Monitoring Unit (CMU).**

The TMC Clinical Monitoring Unit (CMU) is shown in Figure 3. Not all features were necessarily used in this trial.

### Data architecture

This study has complex data management and organisational requirements by virtue of its operation in five different location in five different states and territories, each with their own Ethics requirements and with different hospital systems from which to source hospital data. In this section we describe a secure and effective service-oriented approach for securely managing Telehealth services research data.

The data architecture and data integration services developed as part of this project make a considerable contribution to research and being in the public domain, warrant closer examination. A more detailed description has been published previously [27].

**Figure 4:**
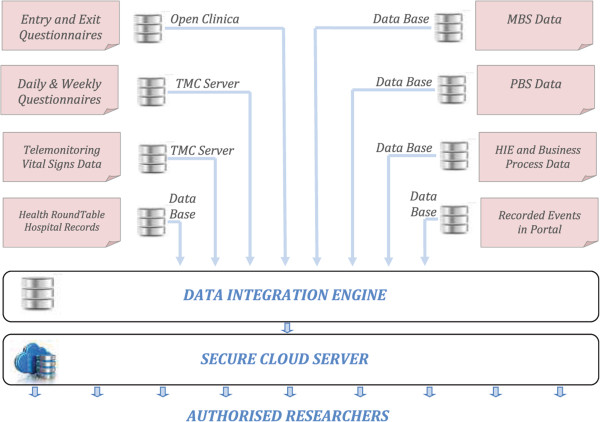
**Schematic diagram of different data sources and their secure integration.**

Data is being collected in this study from many different sources, in multiple formats and with varying levels of automation, with some requiring considerable manual processing. A simplified diagram of data sources used in this study is shown schematically in Figure 4 below.

-  Entry and Exit Questionnaires are administered on line by PO’s when Test and Control patients are consented and are stored in OpenClinica
-  Periodic Questionnaires (daily, weekly or monthly) are scheduled on the TMC clinician website and are presented and administered directly on the patient telemonitoring system. The results are stored in the TMC servers.
-  Patient vital signs are recorded as longitudinal records and original waveforms are recorded and stored in the TMC server for quality control and diagnostic purposes. All records are accessible to the clinicians via the TMC clinician portal.
-  Hospital Data is sourced from the Patient Administration Systems of hospitals servicing the trial sites and is supplied in the format of the Hospital Roundtable [28]. This comprehensive data set is requested for four years prior to enrolment and for the duration of the trial.
-  MBS Data available from the Department of Human Services following Ethics Approval. This provides a comprehensive record of all primary care services provided under the national health insurance scheme
-  PBS Data available from the Department of Human Services following Ethics Approval. This provides a comprehensive record of all medications dispensed under the national health insurance scheme. MBS and PBS Data is available for a total of 4.5 years prior to and including the duration of the trial.
-  HIE Data from focus groups and structured interviews are transcribed and annotated before storage in OpenClinica.

Technical details on the data architecture designed to provide secure Role Based Access Control to de-identified patients data for research and analysis has been previously reported [27].

### Questionnaire instruments

A number of questionnaire instruments were developed or adapted from the literature for use in the trial. All Patients enrolled in the study were required to take an Entry and Exit Questionnaire. This questionnaire instrument was developed from a base CSIRO CAFHS Human Research Ethics Standard Screening Medical Questionnaire [29] with the addition of other questionnaire instruments either wholly or in part, measuring demographic, lifestyle, health and disease characteristics. Key elements of the Entry and Exit Questionnaires are described in Table 3 below.

In addition to the Entry questionnaire, a number of questionnaires are scheduled and administered during the trial with varying frequency. These are described in Table 4 below.

**Table 4:** **Questionnaire Instruments and their schedule**

Questionnaire	Administering schedule
COPD (Developed by the Austin Hospital)	Daily
CHF (Developed by the Austin Hospital)	Daily
EQ-5D (Quality of life)]	Weekly
Kessler 10 (Mental health)	Monthly
heiQ – selected domains (Self monitoring, Health services navigation and Social isolation)	Entry, 6 months, Exit
Morisky medicine adherence scale	Entry, 6 months, Exit

### Data models

As described earlier, patient data is obtained from multiple sources and integrated into a single unified database linked via the unique OpenClinica ID (OCID). A Data Model has been developed which provides the template for data analysis by linking outcomes and objectives to specific data variables and identifying the data sources. This data model underpins nearly all quantitative analysis presented in this report. The data model is presented below in Table 5.

**Table 5:** **Data Model for evaluating outcomes and objectives**

Objective/outcome	Data variable	Data source
Define the study cohort/confirmation of selection critera and exclusions	Admitted to hospital for their condition at least twice in the previous year, or ≥ 4 times in previous five years	Hospital health roundtable records - obtained from local hospital for previous five years.
		• Date admitted
	Exclusions are mental health and cancer patients	• Date discharged
		• Reason for admission (ICD 9/10 Codes)
		• Procedures carried out
Establish if telehealth	Number of unscheduled admissions to hospital for their condition	MBS Flag (In hospital) Health roundtable record
Improves patient outcomes/reduced hospitalisation		• Date admitted
		• Date discharged
		• Reason for admission (ICD 9/10 Classification)
		• Medication administered
		• Procedures carried out
Establish If telehealth improves patient outcomes/reduced use of clinical services (Impact on clinical workforce availability and deployment)	Number of visits to/by GP	MBS records
	Number of visits to/by specialists	MBS records
	Number of visits by community nurse	MBS records
	Number of visits to/by allied health (ie occupational therapist)	MBS records (If reimbursable from Medicare)
	Changes in prescription history	PBS
	Communication with CCC	CCC Logs from CSIRO Portal
Organisational change management and impact on workplace culture	Administrative/operational changes implemented/required in order to implement the Telehealth service.	Questionnaires and structured interviews.
		• Within first three months
		• Every six months thereafter
Useability of monitoring equipment	Compliance with monitoring schedule, recorded daily.	TMC Logs
	Extra measurements taken by patient (When? Which?)	TMC Logs
	Compliance with questionnaire administration (When? Which?)	TMC Logs
	Use of video conferencing	TMC Logs
	Overall data usage	iiNET provided logs
Useability/acceptability	Ease of use	Questionnaires delivered via TMC
For patients of monitoring Equipment	Quality of training received	• One month after first deployment
	Patient embarrassment if visitors know they are being monitored	• Midpoint of trial
	Acceptability as an item of furniture	• At end of trial
	Easy or hard to take measurement	
	Important/not important in patients' self management	
	Responsiveness of clinical care coordinator in responding to changes	
	Quality of training received	
	Patient embarrassment if visitors know they are being monitored?	
	Easy or hard to take measurement	
Carers experience with telehealth (Community nurse/carer)	Ease of use of (i) equipment and (ii) Clinician website	Questionnaires and structured interviews of community nurses
	Changes to previous clinical models of care	• One month after first deployment
	Effectiveness in improving ability to deliver care	• Midpoint of trial
	Impact on workload	• At end of trial
Carer's experience with telehealth (Relative or other carer)	Effect on carer stress	Questionnaires and structured interviews
	Effect on carer workload	• At first deployment
	Effectiveness in improving ability to deliver care	• Midpoint of trial
	Access to clinician web site	• At end of trial
Gp experience with telehealth	Ease of use	Questionnaires and structured interviews of Patients' GP
	Changes to clinical models of care	• Within 3 months of first deployment
	Effectiveness in improving ability to deliver care	• Midpoint of trial
	Impact on workload	• At end of trial
Useability, acceptability of clinician web interface	Ease of use?	Questionnaires and structured interviews
	Quality of training received	• One month after first deployment
	How many hours required	• Midpoint of trial
	Value and ease of use of Video conferencing	• At end of trial
Health economic outcomes	Daily cost of hospitalisation	Health roundtable data
	Cost of procedures carried out whilst in hospital	Health roundtable data
	Cost of visits to/by GP	MBS Data
	Cost of visits to/by Allied Health (ie Chiropodist or OT)	MBS Data
	Cost of visits by community Nurse/carer	MBS Data
	Cost of travel to GP	MBS Data
	Loss of earnings if patient is still employed, from days taken off for illness or visits to health professionals	Use Google Maps to determine distance travelled from home address to address of service location, then apply standard costing model. Ie flag fall + km charge.
		Estimate from patient salary and time spent on each visit
Cost of delivering telehealth services	Cost of clinical care coordinator(s)	Health service provider and logs recorded
	Cost of clinical nurses/carers	Health service provider and logs recorded
	Cost of providing network services	iiNET billing at commercial rates
	Cost of providing Telehealth monitoring services	TMC commercial daily subscription costs
	Depreciated costs of capital equipment	Our own project records
	Estimate of cost of space for monitoring centre at each site	Estimates from Health service Provider

### Data analysis

Data will be analysed using a range of conventional statistical methods used in bio-statistics as well as model based methods more commonly found in the scientific statistical literature.

The independent samples t-test is commonly used when two separate sets of independent, randomly selected and normally distributed samples are available, one from each of the two populations being compared. However as explained previously, our project design makes random selection of Test and Control patients impossible and the alternative Before and After Control Intervention (BACI) design was adopted [22]. This design involved the site PO selecting the test patient first and then selecting from the remaining eligible test patients the controls that best match each test patient in turn.

As a consequence statistical comparisons in this study can only be validly made on Test – Control matched pairs and tested using the paired samples or repeated measures t-tests. We expect to obtain data from 125 test patients and up to 250 control patients. When two or more matched Control patients are available their data is averaged.

Data will be routinely tested for normality and if not normal, the appropriate transform, often log functions, will be applied to normalise the data prior to applying paired samples or repeated measures t-tests. Baseline characteristics will be described for time intervals as mean ± SDs for continuous symmetrical variables and means and 95% Confidence Intervals (CI) for skewed data. When before intervention data are available on outcomes, then the differences between the test and their controls will be tested to examine if there are any differences in the before intervention period. This process will be examined visually by examining boxplots of the time stamped differences (e.g., differences between the respective monthly variables eg. hospital costs) between test and control patient outcome measures.

As is common in the life sciences our data may be lognormal when not zero and may contain numerous zero values. In these circumstances the data will be modelled using a lognormal distribution for positive values, together with an additional probability mass at zero. This type of distribution is commonly referred to as delta-lognormal. 95% Confidence limits are calculated according to the method of Zou, Taleban and Huo [37].

All statistical tests for the before intervention period will be two-sided but all tests of whether the intervention made a difference will involve a one-tailed matched pair t-test, and either a p value of 0.05 or less will be used to indicate statistical significance or a confidence interval will be provided to judge the impact of the intervention. Statistical analysis will be performed using Stata Release V.12 (TX: StataCorp LP), SPSS 17, R package [38] and Microsoft Excel.

### Classical BACI designs and extensions

Because of the temporal nature of health data and the underlying trends caused by the increasing burden of chronic disease with increasing age, before and after comparisons can be difficult or inconclusive. In this study we will have available patient data for a period of 4.5 years including the period of the intervention which may vary from 6–18 months.

Since standard BACI design cannot be used when study variables trend over time, we adopt a mathematical approach using a mixed linear effects model (*lme*) to assess the intervention while trying to adjust for the time varying mean in simple ways. The *nlme* library in the statistical package R is used to perform the analysis according to the methods of Pinheiro et al. [39].

The Test patients will be matched with one or two controls and the relative movement of Test patients’ outcomes before and after the start of the intervention will be compared to that of their Control patients. The before-and-after comparisons use control patients to adjust for background variations in such variables as public health policy impacting on costs and even for example, climatic conditions during the before and after intervention period.

Let *y*_*ijk*_ be any of the variables to be analysed, such as for example, the PBS/MBS/Hospital costs value per unit time period (month) at time k during period *i* (before or after the intervention), for patient *j* (control or intervention patient). The model for the response value is given by


where:

-  *μ* is the overall mean
-  *α*_*i*_ is the effect of period (before and after)
-  τ_k(i)_ is the repeated measures within periods (assumed to be a random effect)
-  β_j_ is the effect on jth matched patients (intervention or control)
-  (αβ)_ij_ is the interaction between period and matched patient groups
-  θ is the overall slope relating to time in months if no differentiation is made between the groups (B-A, C-I)
-  θ_ij_ is the slope relating to time (k) in months for the various combinations of the groups (B-A, C-I).
-  e_ijk_ is the random error term of the model that is assumed to be normally distributed with homogeneous variance.

Assumptions made:

-  Log of cost plus one will be treated as normally distributed with log of the number of days in the month as the offset. Sometimes the square root transformation may be used to stabilise the variance. We are hoping there are not too many zero cost periods or zero counts. If this fails we will use the zero adjusted inverse Gaussian distribution for the model – fitting them using the **gamlss** (package in R) using random() for including random effects [40].
-  τ_k(i)_ is a random effect in the above model that is assumed to be normally distributed with mean zero and constant variance.
-  The assumption in the previous dot point and the assumption for e_ijk_ in the model thus assumes that measurements made at the same time segments (e.g., on the same quarter) have the same correlation and homogeneous variances for all repeated measures.
-  The above model treats the study as a fully-designed experiment with the appropriate randomisation. However, this is seldom the case because most impact studies are observational in nature.
-  The assumption is that each measurement for the intervention patients is matched with a measurement for one or more control patients. This blocking is expected to control for the non-randomisation in the design. The complexity of this analysis can be greatly reduced by taking the differences between the Test and Control measurements
-  The model above tests whether a significant change has occurred by testing the significance of the interaction term of the model for the before after indicator variables and the control-intervention indictor variable. For example if the coefficient for intervention patients and after intervention duration has lower insured costs that before the intervention after adjusting for controls, then the intervention has had a significant impact on costs.
-  The random effects terms and random error term are assumed to be uncorrelated in time.
-  The control patient is generally selected to control for all covariates. In this study this means that control patients should be identical to the intervention patient in terms of age, gender, SEIFA index and major comorbidities.
-  The samples are selected over time (therefore they are time series rather than repeated measures made at the same time). So it may seem unlikely that the model errors will be independently distributed but hospital costs are measured a month apart and this should be enough to for the assumption of independence to be valid.
-  The assumption that all repeated measures have the same variance is unlikely to be true. If the gamlss package is used then this change in variance can be accounted for. Although theoretically longitudinal data structures can be modelled by random effects in gamlss [40] but at present no computationally feasible implementation for large sample sizes and complex models exists.
-  Assume that the trend in the response variable over time is approximately linear. This assumption is likely to be reasonable over the 5 year study period, but is unlikely to be true for longer time periods.

These models will be fitted using the nlme package in R [39].

**Figure 5:**
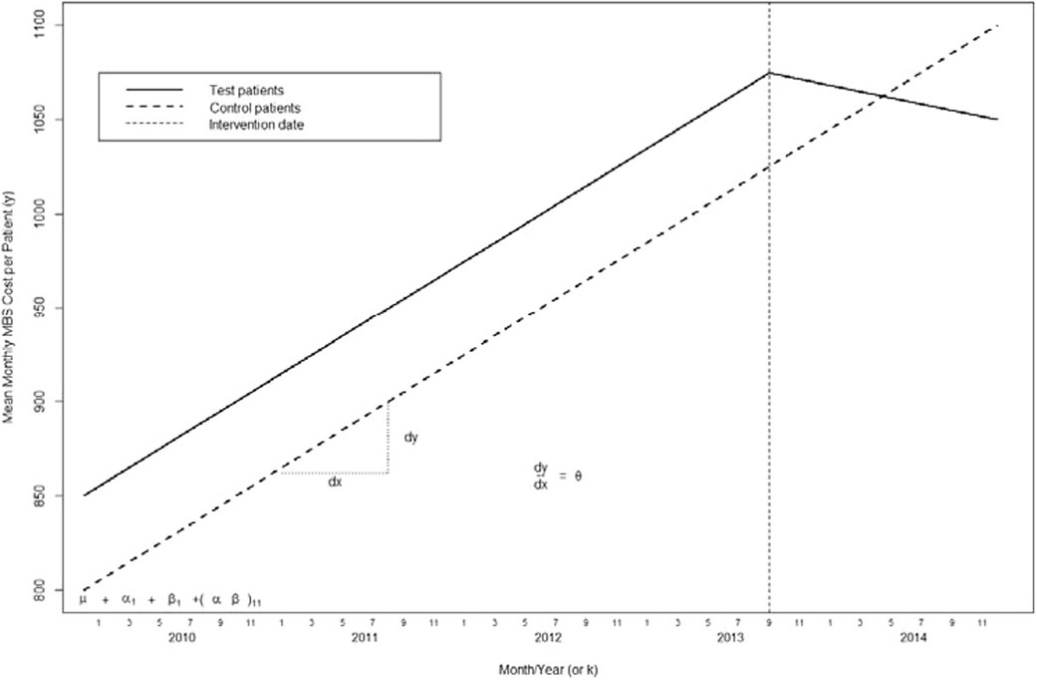
**Output of lme modelling of mean monthly MBS cost per patient for test and control.** Patients based on hypothetical data. Each monthly data point is plotted as a box and whiskers plot representing data for the 125 Test patients and 250 Control patients.

A hypothetical scenario is modelled schematically in Figure 5 below according to principles and assumptions outlined above.

The BACI analysis will be carried out using the statistical modelling package R [38]. The output will provide an estimate of the correlation between Test and Control data and will provide best fits, standard errors, t-values and p-values for all model parameters before and after the intervention.

In the schematic diagram above we would expect very little difference between Test and Control patients in either the slope or the intercept of the model prior to the start of the intervention. The hypothetical model in Figure 5 shows that the impact will result in a change of the historical slope of the model before the intervention for Test patients, representing a time dependent fall in Mean Monthly MBS Costs, whilst there is no corresponding change in the slope for Control patients after the intervention.

Modelling of the available data will reveal underlying time dependent trends associated with the ageing process as well as the impact of the intervention and will allow an estimate to be made of savings associated with the impact of the intervention.

### Power calculations

The power of the tests in the linear mixed model is not easy to compute. The power of a match paired t-test is estimated assuming a correlation of ρ and a standard deviation of σ for the differences in match scores, a decision boundary for a test of size κ departure between the match scores, and no autocorrelation with an effective sample size of 30.

The power calculations based on independent observations and the outcomes of the test are given in Table 6: taking

**Table 6:** **Power calculations for some selected variables**

Outcome measure all on the monthly scale	Effective sample size?	Assumed normal distribution	Shift amount (K)	SPower
PBS Total cost	30	Log(PBS Total cost+1)	1	1.00
MBS out of hospital costs	30	Log(MBS out of hospital costs+1)	1	1.00
MBS in hospital costs	30	Log(MBS in hospital costs+1)	1	0.84
Number of hospital admissions	30	Square root the number of hospital admissions	0.5	0.99
Number of GP visits during working hours	30	Square root of number of GP visits during working hours	0.5	0.89
Number of GP visits outside of working hours	30	Square root of number of GP visits outside of working hours	0.1	0.50
Total number of GP visits	30	Square root of total number of GP visits	1	0.97
Total number of either Specialist, Psychiatric, Allied Health visits and Procedures	30	Square root of total number of either Specialist, Psychiatric, Allied Health visits and Procedures	1	0.77
Total number of Laboratory tests	30	Square root of total number of Laboratory tests	1	0.97
Number of Laboratory	30	Square root of number of Laboratory	1	0.96
Tests		Tests		

The actual results may be much more complicated than this because the differences between the outcome variables may be auto correlated. This is particularly true if the test patient and the matched control patient outcome measures have different time series trends. Testing of whether the matched differences are auto correlated will be carried out when data becomes available but is not expected to be a significant problem.



The actual results may be much more complicated than this because the differences between the outcome variables may be auto correlated. This is particularly true if the test patient and the matched control patient outcome measures have different time series trends. Testing of whether the matched differences are auto correlated will be carried out when data becomes available but is not expected to be a significant problem.

## Discussion

There are many clinical benefits associated with remote patient monitoring with a large range of chronic conditions [9]. Some of the evidence for this includes an increase in mean survival time in a sample of 387 diabetic patients who undertook daily monitoring of vital signs [41], a significant improvement in glycemic control in diabetic patients who transmitted blood glucose and blood pressure data to a telehealth nurse [42], a 71% reduction in Emergency Room (ER) admissions in respiratory patients who had oxygen saturation measured by pulse oximetry and monitored daily [43], a reduction in the number of hospital readmissions in patients with angina [44], significant improvements in health related quality of life and a decrease in mortality in COPD patients using home monitoring [45], a 43% reduction in hospitalizations and a 68% reduction in bed days of care in cardiac patients who transmitted daily ECG and blood pressure data [46] and a 50% reduction in the risk of heart failure related readmission and 55% reduction in cardiovascular mortality in chronic heart failure patients monitored at home [8].

The evidence therefore appears overwhelming that at home telemonitoring can deliver significant patient health benefits at lower cost and with a high level of acceptance by patients and their carers. Deployment of telehealth services however is far from widespread. Broadly speaking telehealth services has been embraced most enthusiastically in the US with uptake in Australia and the rest of the western industrialised nations patchy, tentative and on a small scale rarely proceeding past the trial stage.

Outside of the USA, the United Kingdom has the most evolved infrastructure and government policy framework for supporting at home telemonitoring, and is now promoting a Public-Private Partnership to deploy telehealth services to three million chronically ill patients. In Australia, Government has been preoccupied with the funding of national eHealth infrastructure through the establishment of the National eHealth Transition Authority (NeHTA) [47], and with the development of the national Personally Controlled Electronic Health Record (PCEHR) [48] which is now being slowly deployed and is receiving limited acceptance from clinicians.

Telehealth video consultations between specialists and patients in Residential Care Facilities or remote area community health services are now being funded through the Medicare system and the Consumer Directed Care Program [21] which is replacing the existing Federally funded care packages known as Home and Community Care Packages (HACC), Community Aged Care Packages (CACP) and Extended Aged Care in the Home (EACH), also has provision for the supply of at home tele-monitoring services.

With these initiatives in place it is probable that Australia, will begin to implement large scale at home telemonitoring services over the next few years. However there are significant uncertainties and impediments that need to be resolved before large scale deployment of telehealth services will become routine. These include the following;

-  Concern over funding models. The National Health Insurance system has historically funded provider – patient clinical consultations. There are concerns that telehealth services may lead to cost blowouts in essentially uncapped federal and state healthcare budgets.
-  State and Federal Government cost shifting. In Australia the Federal Government funds primary care and aged care and the State Governments fund hospital services. If the Federal Government funds telehealth to reduce unnecessary hospitalisation of those with chronic conditions, the primary beneficiaries will be the state governments. Hence there is a mis-alignment of those that pay and those that benefit!
-  Limited awareness and support for telehealth services among clinicians, service providers and patients.
-  Varying levels of organisational readiness within State Governments, local health districts and not for profit health service providers for the deployment of telehealth services.
-  A lack of data on how to identify those patients that would benefit most from at home telemonitoring for their chronic conditions, and a robust process for allocating tele monitoring resources throughout the disease life cycle from early intervention for early stage disease conditions such as Type II diabetes, through to complex chronic conditions with multiple co-morbidities such as CHF patients with COPD and CHD.
-  A robust process for selecting competitive at home telemonitoring services that provide the best quality patient data and opportunity for clinical diagnosis. Ensuring that systems are inter operable and standards based and can automatically transfer data securely to either provider controlled or national electronic health records.

The clinical trial design for this national trial was formulated to provide statistically robust evidence, valid across a number of existing healthcare settings in Australia to inform Government policy and funding frameworks as well as to provide a template for the adoption of telehealth services by a range of private and public healthcare service delivery organisations.

## Abbreviations

ADSL: Asymmetric digital subscriber line
ADSL2: Asymmetric digital subscriber line - 2^nd^ specification
CCC: Clinical Care Coordinator
CDM: Clinical Data Management
CHD: Coronary heart disease
CHF: Congestive heart failure
CMU: Clinical Monitoring Unit
COPD: Chronic obstructive respiratory disease
CSIRO: Commonwealth Scientific and Industrial Research Organisation
CTC: Clinical Trial Coordinator
EDC: Electronic data capture
HREC: Human Research Ethics Committee
LHD: Local Health District
MBS: Medical benefits scheme
NBN: National broadband network
PAS: Patient administration system
PBS: Pharmaceutical benefits scheme
PCEHR: Personally controlled electronic patient record
PO: Project Officer
SEIFA: Socio-economic indexes for areas
TMC: Telemedcare telemonitoring devices/services
VDSL: Very-high-bit-rate digital subscriber line.
